# Spatial Distribution, Potential Risks and Source Identification of Heavy Metals in the Coastal Sediments of the Northern Beibu Gulf, South China Sea

**DOI:** 10.3390/ijerph191610205

**Published:** 2022-08-17

**Authors:** Changping Yang, Liangming Wang, Yan Liu, Binbin Shan, Dianrong Sun

**Affiliations:** Key Laboratory of Marine Ranching, Ministry of Agriculture and Rural Affairs, South China Sea Fisheries Research Institute, Chinese Academy of Fisheries Sciences, Guangzhou 510300, China

**Keywords:** heavy metals, distribution, ecological risks, sources, Beibu Gulf

## Abstract

Thirty samples of surface sediments (0–5 cm) from the northern Beibu Gulf were analyzed to determine the spatial distribution, potential risks and sources of six heavy metals (Cr, Cu, Zn, As, Cd and Pb). The concentrations (mg/kg, dw) of Cr, Cu, Zn, As, Cd and Pb were 15.38 ± 6.06, 6.54 ± 3.23, 41.86 ± 17.03, 6.92 ± 2.75, 0.04 ± 0.02 and 17.13 ± 6.38, respectively. Higher levels of Cr, Cu, Cd and Zn were observed in the western part of the study area. According to the potential ecological risk indexes and sediment quality guidelines, the measured metals were assessed at low contamination levels, with Pb posing the largest ecological risks. The results of positive matrix factorization (PMF) indicated that Cr and Zn mainly originated from natural geological background sources, while Cu, As, Cd and Pb were influenced by anthropogenic sources such as atmospheric deposition and anthropogenic activities. These three sources contributed 60.4%, 28.1% and 11.5% of the heavy metals, respectively. In addition, further research should be conducted focusing on the general relationships between As and various controls in sediments of the northern Beibu Gulf.

## 1. Introduction

Heavy metals are pollutants of particular concern due to their bio-toxicity, bioaccumulation, persistence, abundance, and ubiquity in the environment, and subsequent impact on both biotic and abiotic aquatic ecosystems in different ways [1,2]. Heavy metals originate from various sources, such as anthropogenic activities and natural processes [3]. The marine/estuarine surface sediments are important sinks for heavy metals and have been considered as good indicators of heavy metal contamination for its hydrophobicity and accumulation tendency in sediment [4,5]. Sediment resuspension caused by changes in environment conditions such as pH, salinity and organic matter may lead to releasing of heavy metals back into the surrounding water [6]. This will subsequently result in secondary metal distribution and pollution, and ultimately threatens aquatic organisms and human health through bio-assimilation and bioaccumulation in the food chain [7]. Therefore, it is important to understand the spatial distribution and sources of heavy meals in sediments to develop the potential ecological and biological risks of aquatic ecosystems.

The Beibu Gulf is a semi-enclosed bay covering a sea area of 128,000 km^2^, with plenty of seafood and fishery resources. It lies in the northwestern part of South China Sea and is surrounded by Hainan Island, Leizhou Peninsula, Guangxi Province and northern Vietnam [8]. In recent years, the fast development of industry, aquaculture and agriculture around the gulf results in large amounts of industrial effluent and municipal domestic sewage being discharged into the waters [9]. Many rivers flow into the Beibu Gulf, such as Beilun, Nanliu, Dafeng and Changhua Rivers from China and Red River from Vietnam, contributing more than 4 × 10^7^ ton sediment load per year [10,11]. These rivers may inevitably transport organic and inorganic materials into the Beibu Gulf, as well as heavy metal pollutants. Various studies focusing on heavy metal pollution in the intertidal sediments, inner bays and estuary area of the Beibu Gulf have been conducted over the last few decades [8,9,10,11,12], and most of the research results showed that the environmental contamination and degradation around Beibu Gulf have gradually increased due to the rapid development of the local economy. However, heavy metal contamination in sediments can be affected by various surrounding physicochemical factors, which may vary spatially and temporally. The most recent study related to this topic was conducted by Gan et al. (2013) [9], who only studied three trace metals (As, Cd and Hg) in the coastal wetland sediment of the northern Beibu Gulf and found a potential ecological risk of As. Despite this, information regarding the heavy metal distribution and source across the coastal areas of northern Beibu Gulf are still limited and need to be further explored. Therefore, it is necessary to determine the pollution characteristics and sources of heavy metals, as well as assess their potential risks and impacts on the ecosystem in the coastal sediments of northern Beibu Gulf.

Appropriate geochemical approaches, such as geoaccumulation index (*I_geo_*), potential ecological risk index (*RI*), contamination factor (*CF*) and sediment quality guideline method (SQGs) have been successfully applied for evaluation of the sediment quality [13,14,15,16]. Moreover, positive matrix factorization (PMF) is increasingly used for environmental studies, including the source identification of heavy metal pollutants [17]. In the present study, the above-mentioned methods were employed in contamination status and sources of heavy metals in sediments of the northern Beibu Gulf. The major objectives are: (1) to determine the concentrations and distribution patterns of heavy metals (Cr, Cu, Zn, As, Cd and Pb) based on a geographic information system; (2) to evaluate the potential ecological risks associated with metal contaminations using geoaccumulation index and potential ecological risk index; (3) to explore their natural and anthropogenic contributions using a positive matrix factorization model.

## 2. Materials and Methods

### 2.1. Study Area and Sampling Method

**Figure 1:**
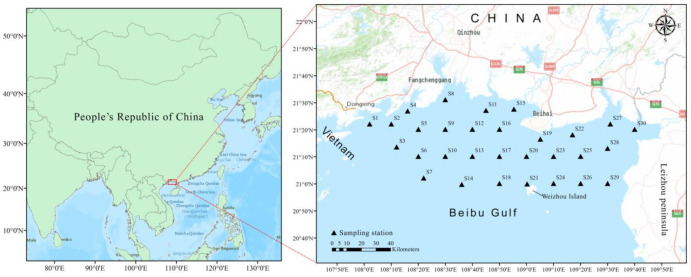
Sampling sites and location in the northern Beibu Gulf.

In September 2018, a total of 30 surface sediment samples were obtained from the northern coastal area of Beibu Gulf using a Van Veen grab sampler (Figure 1). All sampling stations were selected carefully to make good area coverage. The surface sediments (0–5 cm) were collected using a polyethylene scraper. Three replicate sediments of each station were well mixed and then encapsulated in acid-rinsed plastic packages immediately. After collection, the surface sediment samples were transported to the laboratory and stored at −20 °C for further analysis.

### 2.2. Analytical Methods

All the sediment samples were freeze-dried, ground, and then sieved through a 0.5 mm nylon mesh to remove large debris. Approximately 0.15 g samples were microwave-digested in acid-washed Teflon digestion vessels. Samples were treated with a mixed solution of 2 mL HNO_3_, 6 mL HCl, and 1 mL HF at 190 °C for approximately 30 min. The digestion procedure was performed using a microwave digestion system (Ethos UP, Milestone, Sorisole, Italy). The treated samples were diluted to a specified volume after adding 5% HNO_3_. Then, the concentrations of five heavy metals (Cr, Cu, Zn, Cd and Pb) were determined using an inductively coupled plasma mass spectrometer (ICP-MS) (ICP-MS, Perkin Elmer ELAN 9000/DRC-e, Shelton, CT, USA), while concentrations of As were examined using the method of atomic fluorescence spectrometry (AFS). Contents of total organic carbon (TOC) were analyzed using the potassium dichromate oxidation-colorimetric method. All of the concentration values in the present study were presented in mg/kg dry weight.

For strict quality assurance and quality control, reagent blank, procedural blank samples, and Chinese National Reference Materials ERM-S-510204 were applied throughout the study. Duplicate samples were examined three times and the relative deviations were below 7%. The results of reference materials were consistent with the certified values, being 108%, 115%, 96%, 109%, 91% and 93% for Cr, Cu, Zn, Cd, Pb and As, respectively. All reagents were prepared with 10% HNO_3_ and Milli-Q water to eliminate possible contamination.

### 2.3. Assessment of Heavy Metals

#### 2.3.1. Spatial Analysis

Spatial analyzing methods based on geographic information system have been commonly used to map and investigate environmental contamination [18,19,20]. In the present study, ArcGIS 10.2.2 software (ESRI, Redlands, CA, USA) was employed to display the spatial distributions, variation trends and contamination levels of heavy metal contents in the sediments. The inverse distance weighted (IDW) module under Spatial Analyst Tools was applied as a predictive approach.

#### 2.3.2. Sediment Quality Guideline (SQG)

In natural environments, human activities may affect multiple contaminations, and sediment quality guidelines (SQGs) were used to determine the potential harmful effects on aquatic environments. Two metrics, threshold effect level (*TEL*) and effect range median (*ERM*), were selected to classify the pollution degree in this method. The corresponding *TEL* and *ERM* values are listed in Table S1. The mean-effect range medium-quotient (m-ERM-Q) is a useful tool to assess the combined ecological effects of different metals [21], and can be calculated with the following relationship:

$$m-\mathrm{ERM}-Q=\overset{n}{\underset{i=1}{\sum }}\frac{C_{i}}{ERM_{i}}/n$$

where *C_i_* is the concentration of metal *i* in sediment, *ERM_i_* is the ERM value for the selected metal *i* and *n* is the number of measured metals. Generally, the SQGs can be categorized into seven classes [21,22], which are presented in Table S2.

#### 2.3.3. Geo-Accumulation Index (I_geo_)

The geo-accumulation index (*I_geo_*) has been frequently used to assess the contamination degree of heavy metals by reducing the impact of geological contributions [23], which can be calculated using the following formula:

$$I_{geo}=log_{2}\left(C_{i}/1.5\times B_{i}\right)$$

where *C_i_* is the metal concentration in sediment and *B_i_* is the geochemical background value of metal *i*. In this study, we use the background values according to Zhang and Du (2005) [24]. A factor of 1.5 is used to minimize the impact of any variations in the background data. The categories of *I_geo_* are divided into seven classes (Table S3).

#### 2.3.4. Potential Ecological Risk Index (*RI*)

Firstly proposed by Hakanson (1980) [25], the potential ecological risk index (*RI*) was a useful approach for determining the unique and combined contamination risks caused by heavy metals. In this approach, not only the contamination level of each metal element can be evaluated, but also the ecological and environmental factors combined with toxicological risks are taken into consideration [26,27]. *RI* can be calculated with the equation:

$$RI=\overset{n}{\underset{i=1}{\sum }}E_{r}^{i}=\overset{n}{\underset{i=1}{\sum }}T_{r}^{i}\cdot \frac{C_{s}^{i}}{C_{n}^{i}}$$

where *E_r_^i^* is the potential ecological risk index of heavy metal *i*, *C_s_^i^* is the content of element *i* in sediment sample, *C_n_^i^* is the background value of element *i*, *T_r_^i^* is the toxic and biological response factor of metal *i*. The *T_r_^i^* values for Cr, Cu, Zn, Cd, Pb and As are 2, 5, 1, 30, 5 and 5, respectively [28]. The calculated *E_r_^i^* and *RI* values are then categorized into five and four grades, respectively, which are given in Table S4.

#### 2.3.5. Positive Matrix Factorization (PMF)

The PMF method was firstly proposed by Paatero and Tapper (1994) [29] and has been considered as an advance receptor model to identify pollutant sources in a natural environment [30]. In this study, PMF 5.0 (US EPA, 2014) [31] was applied to apportion the contributions from emission metal sources in the sediments since it has several advantages over principal component analysis (PCA) [32,33]: (1) there is no non-negativity constraint; (2) the factors are not orthogonal to each other; (3) the data point is uncertainty-weighted. Thus, the PMF method can avoid negative false loading values of each factor and provide a good interpretation of results.

The matrix *X_ij_* can be viewed as an ambient data set in which *i* and *j* represent the number of samples and the number of metal species, respectively. The multivariate receptor modeling has three goals: (1) to identify a number of metal sources (*p*), (2) to distinguish the species profile (*f*) of each source, (3) to assess the amount of mass (*g*) contributed by each source species to each sediment sample as well as the related residual (*e_ij_*). *X_ij_* can be expressed using the following relationship:

$$X_{ij}=\overset{p}{\underset{k=1}{\sum }}g_{jk}f_{kj}+e_{ij}$$

where *e_ij_* represents the related residual of each sediment sample or source species.

In the PMF model, the different number of sources was tested to find out the optimal value corresponding to the most reasonable results. The properly estimated source number indicated the theoretical Q value was approximately the number of degrees of freedom or the total number of data points. Based on the uncertainties (u), the PMF solution minimizes the objective function Q as the following relationship:

$$Q=\overset{n}{\underset{i=1}{\sum }}\overset{m}{\underset{j=1}{\sum }}\left[\frac{X_{ij}-\sum_{k=1}^{p}g_{ik}f_{kj}}{u_{ij}}\right]$$

Two data files including metal concentrations and uncertainty data were input to run the PMF model. According to Zhang et al. (2014) [30], 15% of the measured metal concentrations were taken as the uncertainty matrices. The model was run 20 times with 25 random seeds to evaluate the stability of goodness-of-fit values. The rotational freedom parameter function in the PMF 5.0 model can control whether more extreme values are assumed for the factor loadings or scores. In the present study, Q (Robust) and Q (True) were 7764.9 and 7628.5, respectively. Calculated with PMF, approximately 98% of the scaled residuals ranged from −3 to 3, suggesting a good fit of the modeled results.

#### 2.3.6. Statistical Analysis

In the present study, the data normality was evaluated using the Kolmogorov–Smirnov test, and all the data showed normal distribution. Pearson correlation analysis (PCA) and Pearson Correlation Matrix (PCM) were applied to determine the relationships among heavy metals in the surface sediments. The statistical analysis was performed with the statistical package SPSS 19.0 (SPSS Inc., Chicago, IL, USA). Hierarchical clustering analyses (HCAs) of heavy metals and sampling stations were conducted using the Gplot package in R software [34].

## 3. Results

### 3.1. Metal Levels and Distributions in Surface Sediments

**Figure 2:**
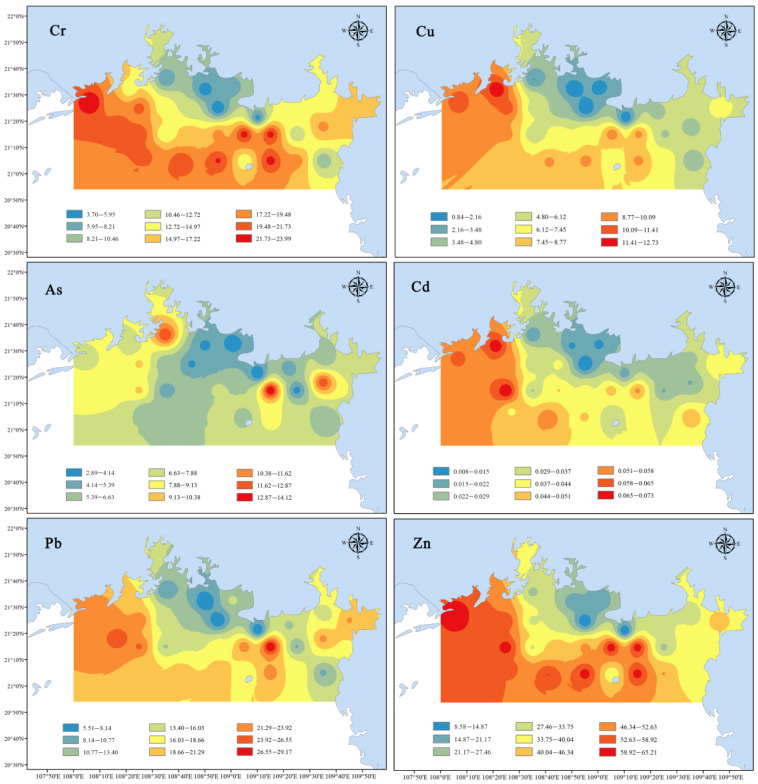
Spatial distribution of heavy metals (mg/kg, dry weight) in surface sediments of the northern Beibu Gulf.

The concentrations and distribution patterns of six heavy metals (Cr, Cu, Zn, As, Cd and Pb) in sediments from Northern Beibu Gulf are summarized in Table 1 and Figure 2, respectively. The concentration ranges of the studied metals are 3.70–24.00 mg/kg, 0.84–12.74 mg/kg, 8.58–65.23 mg/kg, 2.89–14.13 mg/kg, 0.01–0.07 mg/kg and 5.51–29.19 mg/kg for Cr, Cu, Zn, As, Cd and Pb, respectively, generally decreasing by the order of Zn (41.86 ± 17.03 mg/kg) > Pb (17.13 ± 6.38 mg/kg) > Cr (15.38 ± 6.06 mg/kg) > As (6.92 ± 2.75 mg/kg) > Cu (6.54 ± 3.23 mg/kg) > Cd (0.04 ± 0.02 mg/kg). In comparison with the background values of regional ecological geochemical survey of the South China Sea (39.3, 7.43, 54.4, 9.71, 0.18 and 15.6 mg/kg for Cr, Cu, Zn, As, Cd and Pb, respectively) [24], 40%, 33%, 10% and 60% of the sediment samples were detected with metal contents exceeding the reference values for Cu, Zn, As and Pb, respectively.

**Figure 3:**
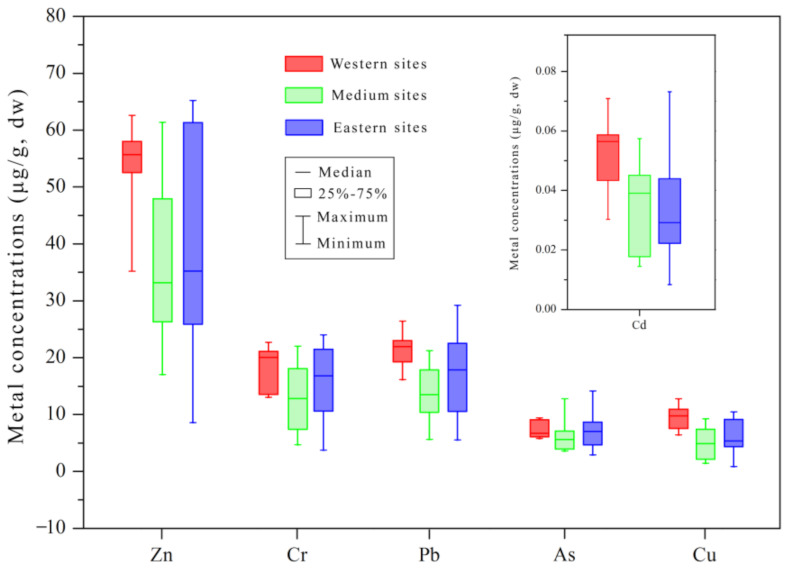
Distribution pattern of heavy metals for three classified station groups of the study region. Western sites, Medium sites and Eastern sites represent station S1–S7, S8–S18 and S19–S30, respectively.

In the present study, some metals like Cr, Cu, Cd and Zn in the western stations were relatively higher than those in the eastern stations (Figure 2). This spatial distribution pattern suggested that the anthropogenically sourced metals might be affected by the circulation characteristics in the northern Beibu Gulf. In order to clearly analyze the spatial distribution of heavy metals, the thirty stations were classified into three groups: western group (S1–S7), medium group (S8–S18) and eastern group (S19–S30). The average metal concentrations decreased by the order of western group > eastern group > medium group for Zn, Cr, Pb, As and Cu, while Cd followed the sequence of western group > medium group > eastern group (Figure 3). All peak values of the examined metals were presented in the western area of the study region except for that of As. Hydrodynamic conditions are important and direct factors influencing the spatial and temporal distribution of heavy metals in the surface sediments [35]. According to Chen et al. (2020), the general counterclockwise circulation water current in the Beibu Gulf and clockwise circulation in the offshore waters from the Fangchenggang eastward to the Beihai led to an increase of siltation in the northwestern part (especially in the Beilun Estuary) [36]. Heavy metals from the land-sourced contaminants around Beibu Gulf are adsorbed by the fine sediment particles, transported by the water flow and then enriched in the northwest region of the gulf. Additionally, Pearson correlation analysis showed that the TOC contents in the sampling stations were significantly correlated with concentrations for each heavy metal (Figure S1), suggesting that organic matter was a good controlling factor in reflecting the geochemical behavior and migration of heavy metals in sediments [37].

**Figure 4:**
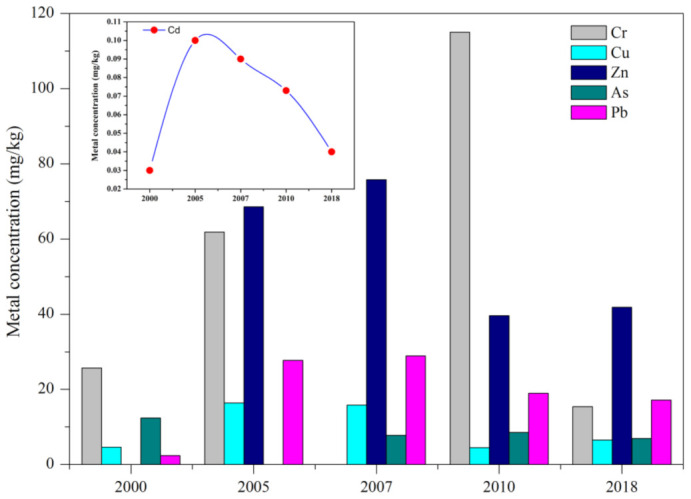
Historical trends (2000–2018) of heavy metals in surface sediments of the Beibu Gulf.

Table 2 compared the metal concentrations of this study with some other sea regions around the world [38,39,40,41,42,43,44,45,46,47,48,49,50]. Concentrations of Cr, Cu, Zn, Pb and Cd in sediments of northern Beibu Gulf were at low levels in comparison with their corresponding background values [24] and those reported in other bay areas except Palau (Korea). Metal contents of most analyzed elements in sediments from Korotoa and Bangshi (Bangladesh) were reported much higher than other regions, mainly due to the large number of anthropogenic inputs (e.g., industrial and agricultural wastes) being discharged into the rivers [47,48]. Meanwhile, significant higher concentrations for Pb were observed in sediments of Jiuzhen Bay (China) whose coast held multiple human activities and industries related to Pb pollution, such as shipbuilding, placer mining and oil refining [39]. In addition, the heavy metal contents of the present study were compared with historical data in the Beibu Gulf since 2000 (Figure 4). Generally, the results showed that concentration levels of the metals reached peak values during 2005–2010 because the Chinese industry was in a stage of rapid development in that period. Since the 18th National Congress of the Communist Party of China, the Chinese government has paid more attention to the environmental and ecological protection. Controlling of pollutant sources, such as mineral mining, metal smelting, petroleum refining and land reclamation might contribute to the low levels of heavy metals in sediments of northern Beibu Gulf.

### 3.2. Sediment Quality Guidelines (SQGs)

Compared with the standard values for the marine sediment quality (GB 18668-2002) of China, the examined heavy metal concentrations of the analyzed elements were all within the range of Grade I (MSQ-1) criteria, indicating good sediment quality in the northern Beibu Gulf. Our results were different from those previously reported by Dou et al. (2013) [51], who found that mean contents of Cr and Cu surpassed the MSQ-1 reference values in the northern part of the Beibu Gulf.

The potential ecological effects caused by heavy metals in the sediments were evaluated by *TEL* and ERM SQGs. The average concentrations of all the six examined metals in sediments of the study region were lower than their corresponding *TEL* values, suggesting no adverse biological effects to the local benthic organisms. Generally, As concentrations in nine sediment samples (30%) exceeded the *TEL* reference values but were lower than the ERM reference values, which indicates occasional occurrence of adverse biological effects. However, As is reputed to be a redox-sensitive element and can respond to redox variation under some conditions due to its nature; thus, its enrichment is often overestimated [52]. Therefore, the general relationships between As and various controls in sediments of northern Beibu Gulf need to be further researched.

**Figure 5:**
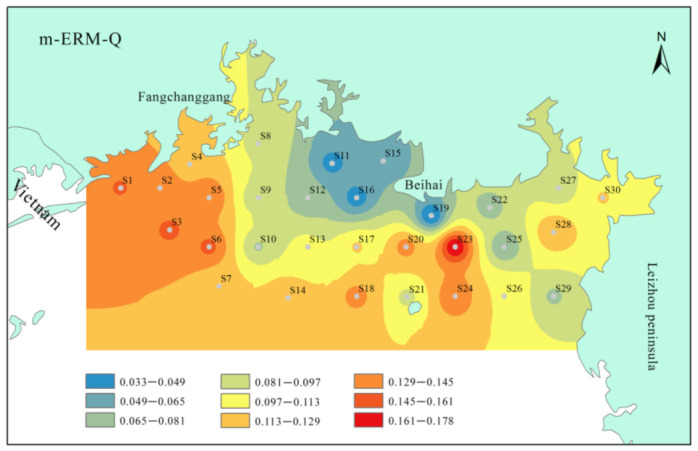
Spatial distribution of m-ERM-Q values in surface sediments of the northern Beibu Gulf.

The spatial distribution of m-ERM-Q values for all sampling stations was illustrated in Figure 5. The values of m-ERM-Q ranged from 0.033 to 0.178, with an average value of 0.107. Generally, the m-ERM-Q values of 17 stations in the study region were higher than the first threshold value (0.1), indicating a 21% probability of negative biological toxicity. Relative higher potential risk stations were found in the southern part of Fangchenggang city (S1–S7) and the northeastern area of Weizhou Island (S23 and S24), suggesting that the sediments in these regions cause higher adverse biological effects than other areas.

### 3.3. Contamination Degree and Risk Assessment

**Figure 6:**
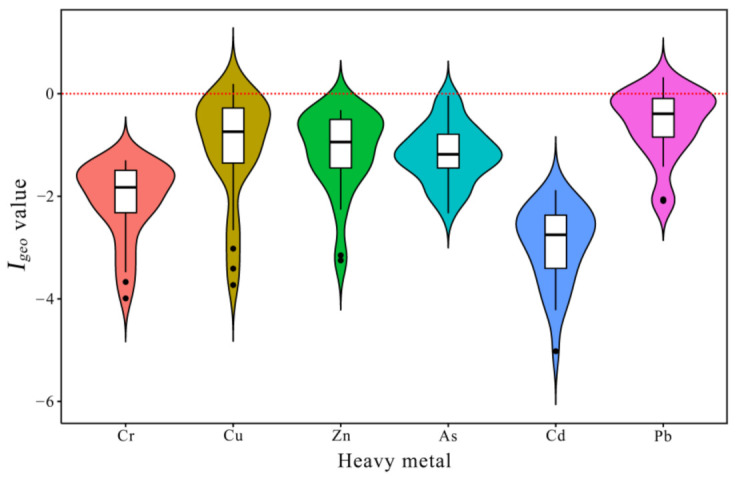
*I_geo_* values of heavy metals in surface sediments of the northern Beibu Gulf.

The geoaccumulation (*I_geo_*) indexes of the six measured heavy metals were calculated based on the Earth’s crust and their values were presented in Table S5 and Figure 6. In general, all sampled sediments exhibited negative *I_geo_* values for Cr, Cu, Zn, Cd and As, and were characterized as “Uncontaminated”. The means *I_geo_* values ranked in the following order: Pb > Cu > Zn > As > Cr > Cd, ranging from −2.95 to −0.58. The *I_geo_* values for the majority of metals except Pb in three stations were lower than 0, indicating that most of the measured elements pose negligible contamination risk in the coastal northern Beibu Gulf. The highest *I_geo_* values for Pb appeared in the station S23, which is located in the northeastern part of the Weizhou oil fields. The long-term and large-scale exploration of petroleum were considered to increase the pollution risk of heavy metals such as As and Pb, and likely pose adverse impacts on the seafloor environment [53]. However, the measured Pb concentrations in this study were far lower than the sediment quality guidelines proposed by the National Oceanic and Atmospheric Administration (NOAA) [22].

In order to assess the potential ecological risks, *E_r_^i^* values for each metal and their *RI* indexes were determined and the related results are given in Table S6. The calculated *E_r_^i^* values were in the range of 0.19–1.22 for Cr, 0.56–8.57 for Cu, 0.16–1.20 for Zn, 1.49–7.28 for As, 1.39–12.20 for Cd and 1.77–9.36 for Pb. The mean *E_r_^i^* values of all the metals were below 40 and followed the order of Zn (0.77) > Cr (0.78) > As (3.56) > Cu (4.40) > Pb (5.49) > Cd (6.56), suggesting that the ecological risks were considered to be low. In addition, the *RI* values were lower than the first threshold (150) in all samples of northern Beibu Gulf, again indicating low ecological risks from these heavy metals. Overall, the values of *E_r_^i^* and RI showed a similar distribution pattern, and were primarily affected by anthropogenic activities and hydrodynamic conditions [51,54].

### 3.4. Sources and Transport of Heavy Metals

**Figure 7:**
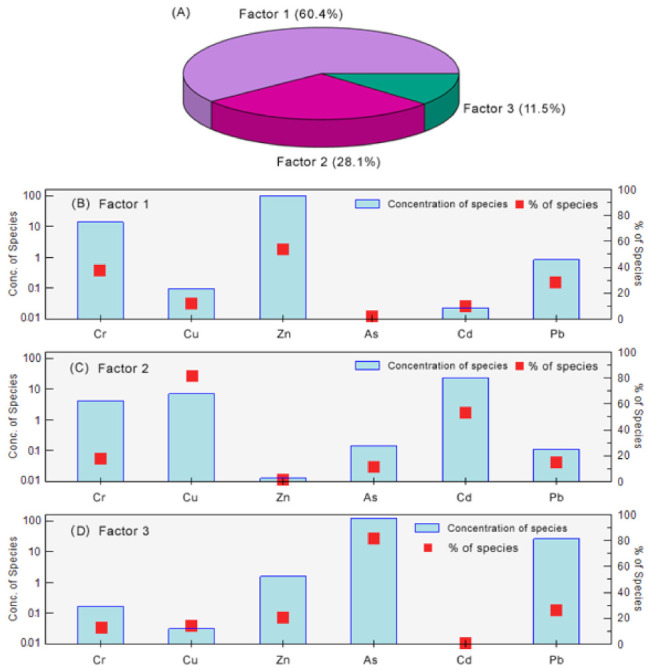
Individual contributions of three major factors (**A**) and factor profiles (**B**–**D**) generated by the PMF model.

In the present study, three main sources of heavy metals were identified using the PMF model and related results are exhibited in Figure 7A. The largest contributing factor was determined as natural geological sources, which contributed 60.4% of the total metal concentrations. Atmospheric deposition and anthropogenic activities accounted for 28.1% and 11.5% of the heavy metals, respectively.

The first source was characterized by Cr, Zn and Pb, and the Pearson correlation coefficients among these metals ranged from 0.905 to 0.942 (*p* < 0.01) (Figure 7B, Table S7). Concentrations of Cr and Zn were lower than their corresponding background values, indicating that these two metals were not affected by anthropogenic activities. In the marine environment, Cr and Zn mainly originate from natural geological background sources such as the diagenetic mobilization of ocean [55]. Meanwhile, the mean Pb content in the sediments was slightly higher than the reference background value, suggesting a potential but low pollution status. The element Pb was considered to be correlated with discharge of oil industries, leaded gasoline, atmospheric deposition, chemical manufacturing and transporting emissions [56,57].

The elements with high contributions to the second source were Cu and Cd, and the Pearson correlation coefficient between these two metals was 0.894 (*p* < 0.01) (Figure 7C). Concentrations of Cu and Cd varied in the study region, with relatively higher values in the west part and relatively lower levels in the northeast part. This distribution pattern might mainly be affected by anthropogenic activities. According to Christophoridis et al. (2019) [46], municipal sewage, effluent industrial discharges and vehicular traffic could be considered as probable sources of Cu. Though Cd concentration was low in the present study, relatively high levels in the coastal area of Fangchenggang city were formerly reported in previous studies [9,10,51], which suggested that Cd originated primarily from inputs from northern rivers including Beilun River and Maoling River.

The profile of the third source was dominated by As (Figure 7D), and its loading profile was different from the other heavy metals. The absence of close correlations between As and the other elements indicated that the anthropogenic source of As was unique. Several previous studies have reported that As in the marine sediments might be attributed to the anthropogenic activities around the northern Beibu Gulf, such as use of copper arsenate [58], arsenic sulfide [59] and arsenical pesticides [60]. In addition, arsenic originated from the natural rocks and organic matter should not be neglected [52], and the exact As source in the study area should be specially discussed in future research.

**Figure 8:**
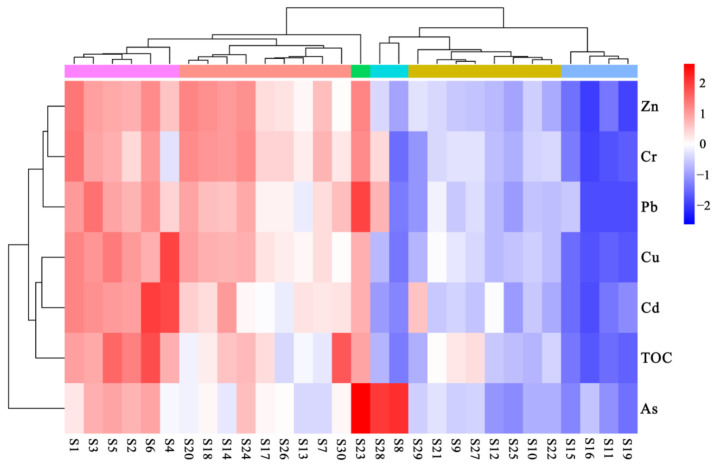
Hierarchical cluster analysis of heavy metals and sampling stations in the northern Beibu Gulf.

Hierarchical clustering analysis results were in consonance with the above findings of PMF analyses (Figure 8). The cluster analysis exhibited that three distinct metal groups were strongly supported: the first includes Zn, Cr and Pb, while the second and third include two metals (Cu and Cd) and one single metal (As), respectively. The strongest association was observed between Zn and Cr, with a similarity larger than 95%.

## 4. Conclusions

In the present study, six heavy metals in the surface sediments of northern Beibu Gulf were measured to investigate their distribution, sources and contamination levels. The measured concentrations were all within the range of Grade I (MSQ-1) criteria according to the marine sediment quality (GB 18668-2002) of China. In comparison with historical data, the metal concentrations were found to be at relatively low levels. The overall spatial distribution pattern was characterized by relatively higher metal levels in the western region. The calculated values of m-ERM-Q showed that Pb and As exhibited higher negative biological effects than the remaining four metals, with the toxic probability occurrence amounting to 21%. *I_geo_*, *E_r_^i^* and *RI* values indicated that the measured elements posed low ecological risks to the seafloor environment. Moreover, our findings suggest that Cr and Zn mainly originated from natural geological background sources, while Cu, As, Cd and Pb were influenced by anthropogenic sources such as industrial discharges, leaded gasoline, atmospheric deposition and agricultural activities.

## Figures and Tables

**Table 1:** Heavy metal concentrations (mg/kg, dry weight) and total organic carbon (TOC, %) in sediments of the northern Beibu Gulf.

Station	Cr	Cu	Zn	As	Cd	Pb	TOC
S1	24.00	10.45	65.23	7.57	0.060	23.68	0.71
S2	17.77	9.77	55.73	9.08	0.056	21.98	0.78
S3	21.09	10.06	58.00	9.10	0.059	26.41	0.68
S4	13.57	12.74	52.55	6.75	0.071	20.01	0.67
S5	20.44	10.90	56.24	9.38	0.057	22.99	0.85
S6	21.46	9.11	62.01	9.41	0.073	24.29	0.92
S7	20.07	7.60	53.43	5.79	0.043	19.28	0.41
S8	6.37	2.13	26.28	12.75	0.018	8.76	0.13
S9	13.67	5.70	32.58	5.54	0.032	13.50	0.52
S10	12.79	4.85	33.16	4.68	0.030	13.14	0.28
S11	4.64	1.37	18.53	3.85	0.015	5.63	0.09
S12	11.51	4.33	29.41	3.92	0.039	12.48	0.32
S13	16.42	6.76	43.00	5.79	0.044	16.00	0.45
S14	21.56	9.05	58.97	6.26	0.057	21.11	0.61
S15	7.39	1.76	16.98	3.54	0.014	13.74	0.12
S16	3.70	0.84	8.58	5.26	0.008	5.51	0.04
S17	18.04	7.42	47.97	7.10	0.039	17.81	0.55
S18	21.97	9.20	61.39	7.12	0.045	21.19	0.51
S19	5.28	1.05	9.22	2.89	0.019	5.53	0.06
S20	22.66	9.70	62.60	6.62	0.048	22.83	0.43
S21	13.04	6.43	35.15	6.12	0.030	16.15	0.48
S22	12.98	4.44	27.50	4.66	0.024	12.96	0.36
S23	22.62	9.23	62.38	14.13	0.053	29.19	0.70
S24	22.54	9.16	61.32	8.69	0.041	22.51	0.64
S25	10.59	4.61	25.87	3.68	0.022	10.54	0.30
S26	18.11	7.07	46.46	7.04	0.036	17.81	0.36
S27	13.48	5.33	31.32	5.74	0.029	15.12	0.54
S28	17.89	4.33	35.24	12.43	0.022	21.84	0.27
S29	8.88	4.16	36.59	5.61	0.050	10.38	0.26
S30	16.79	6.57	42.02	7.17	0.044	21.45	0.89
Range	3.70–24.00	0.84–12.74	8.58–65.23	2.89–14.13	0.008–0.073	5.51–29.19	0.04–0.92
Mean	15.38	6.54	41.86	6.92	0.04	17.13	0.46

**Table 2:** Comparison of heavy metals (mg/kg, dry weight) in sediments of the northern Beibu Gulf with other studies around the world.

Study Region	Cr	Cu	Zn	As	Cd	Pb	Reference
Northern Beibu Gulf, China	15.38 (3.70–24.00) ^a^	6.54 (0.84–12.74)	41.86 (8.58–65.23)	6.92 (2.89–14.13)	0.04 (0.01–0.07)	17.13 (5.51–29.19)	The present study
Yueqing Bay, China	61.5 ± 7.1	34.2 ± 6.4	107.3 ± 0.4	12.3 ± 1.1	0.12 ± 0.10	28.4 ± 2.3	[38]
Jiuzhen Bay, China	na	8.6 (1.1–21)	57 (9–134)	6.3 (3.7–8.9)	0.062 (0.016–0.139)	136 (11–946)	[39]
Mirs Bay, China	29.94 (24–33)	11.63 (8–14)	69.13 (55–76)	6.62 (5.3–7.7)	na	31.5 (26–35)	[40]
Daya Bay, China	59.03 (10–85)	16.46 (1–39.5)	87.81 (13–125)	8.16 (1.94–13.67)	0.07 (0.03–0.13)	37.01 (11–56)	[41]
Coastal Shandong Peninsula, China	57.8 (35.0–99.6)	20.0 (6.7–46.2)	74.7 (37.0–181.1)	na	na	28.4 (19.0–42.2)	[42]
Bohai Bay, China	33.5 (10.2–84.3)	22.7 (7.8–38.6)	71.7 (40.5–126)	na	na	21.7 (8.7–53.6)	[43]
South Yellow Sea, China	na	16.9 (6.0–32.9)	93.7 (24.6–244)	na	na	17.8 (6.2–39.3)	[44]
East China Sea, China	93.5 (45.1–142)	37.4 (18.8–49.2)	116 (80.2–140)	na	na	34.1 (24.4–53.0)	[45]
Thessaloniki Bay, Greece	115.4 (65.5–173.5)	77.23 (21.3–180.1)	218 (141.5–538.2)	na	2.51 (0.2–13)	84.19 (29.4–195.4)	[46]
Korotoa, Bangladesh	109 (55–183)	76 (35–118)	na	25 (2.6–52)	1.2 (0.26–2.8)	58 (36–83)	[47]
Dhaka, Bangladesh	98.1 (42.81–137.04)	31.01 (18.03–51.35)	117.15 (78.42–174.15)	1.93 (0.65–3.15)	0.61 (0.33–0.93)	59.99 (37.48–86.52)	[48]
Palau, Korea	12.0 (1.3–59.1)	8.0 (1.1–66.4)	11.0 (0.7–43.5)	12.8 (0.8–43.5)	0.018 (0.001–0.052)	1.0 (0.1–2.7)	[49]
Funafuti Atoll, Tuvalu	56.7 (26.2–87.6)	23.2 (9.6–38.4)	100 (34.9–204)	na	0.7 (0.4–0.9)	40.9 (20.7–67.2)	[50]

Notes: “^a^”, values in and out the bracket indicate range and mean, respectively; “na” means the related data are not available.

## Data Availability

Not applicable.

## Supplementary Materials

The following supporting information can be downloaded at: https://www.mdpi.com/article/10.3390/ijerph191610205/s1. Figure S1: Linear regression of TOC contents versus logarithm transformed metal concentrations in sediments of the northern Beibu Gulf; Table S1: Sediment quality (SQGs) values for each heavy metal (mg/kg); Table S2: Classification and description of sediment quality guidelines (SQGs); Table S3: Classification and description of *I_geo_* values for heavy metals in surface sediments; Table S4: Classification and description of ecological risk index (*E_r_^i^*) and hazard quotient index (*RI*); Table S5: *I_geo_* values for metals in the sediment samples; Table S6: *E_r_^i^* and *RI* values for heavy metals in surface sediments of the northern Beibu Gulf; Table S7: Pearson correlation (PC) coefficient matrix of heavy metals in the surface sediments of the northern Beibu Gulf.

## References

[1] Sindern S., Tremöhlen M., Dsikowitzky L., Gronen L., Schwarzbauer J., Siregar T.H., Ariyani F., Irianto H.E. (2016). Heavy metals in river and coast sediments of the Jakarta Bay region (Indonesia)—Geogenic versus anthropogenic sources. Mar. Pollut. Bull..

[2] Nguyen B.T., Do D.D., Nguyen T.X., Nguyen V.N., Phuc Nguyen D.T., Nguyen M.H., Thi Truong H.T., Dong H.P., Le A.H., Bach Q.V. (2020). Seasonal; spatial variation; and pollution sources of heavy metals in the sediment of the Saigon River, Vietnam. Environ. Pollut..

[3] Sundaray S.K., Nayak B.B., Lin S., Bhatta D. (2011). Geochemical speciation and risk assessment of heavy metals in the river estuarine sediments—A case study: Mahanadi basin, India. J. Hazard. Mater..

[4] Szefer P., Glasby G.P., Pempkowiak J., Kaliszan R. (1995). Extraction studies of heavy-metal pollutants in surficial sediments from the southern Baltic Sea off Poland. Chem. Geol..

[5] Morris A.W., Allen J.I., Howland R.J.M., Wood R.G. (1995). The estuary plume zone—Source or sink for land-derived nutrient discharges. Estuar. Coast. Shelf. Sci..

[6] Ip C.C.M., Li D.X., Zhang G., Wai O.W.H., Li Y.S. (2007). Trace metal distribution in sediments of the Pearl River Estuary and the surrounding coastal area, South China. Environ. Pollut..

[7] Herut B., Hornung H., Krom M.D., Kress N., Cohen Y. (1993). Trace metals in shallow sediments from the Mediterranean coastal region of Israel. Mar. Pollut. Bull..

[8] Xu Y.Y., Wang Y.H., Li J., Liu X., Zhang R.J., Guo S.J., Huang W.Y., Zhang G. (2013). Distributions, possible sources and biological risk of DDTs, HCHs and chlordanes in sediments of Beibu Gulf and its tributary rivers, China. Mar. Pollut. Bull..

[9] Gan H.Y., Lin J.Q., Liang K., Xia Z. (2013). Selected trace metals (As, Cd and Hg) distribution and contamination in the coastal wetland sediment of the northern Beibu Gulf, South China Sea. Mar. Pollut. Bull..

[10] Gu Y.G., Huang H.H., Liu Y., Gong X.Y., Liao X.L. (2018). Non-metric multidimensional scaling and human risks of heavy metal concentrations in wild marine organisms from the Maowei Sea; the Beibu Gulf, South China Sea. Environ. Toxicol. Phar..

[11] Xu D., Wang R., Wang W.G., Ge Q., Zhang W.L., Chen L., Chu F.Y. (2019). Tracing the source of Pb using stable Pb isotope ratios in sediments of eastern Beibu Gulf, South China Sea. Mar. Pollut. Bull..

[12] Yang C.P., Yu G., Liu Y., Shan B.B., Wang L.M., Sun D.R., Huang Y.B. (2022). Heavy Metal Distribution in Surface Sediments of the Coastal Pearl Bay; South China Sea. Processes.

[13] Schiff K.C., Weisberg S.B. (1999). Iron as a reference element for determining trace metal enrichment in Southern California coastal shelf sediments. Mar. Environ. Res..

[14] Villaescusa-Celaya J.A., Gutierrez-Galindo E.E., Flores-Munoz G. (2000). Heavy metals in the fine fraction of coastal sediments from Baja California (Mexico) and California (USA). Environ. Pollut..

[15] MacDonald D.D., Ingersoll G.C., Berger A.T. (2000). Development and evaluation of consensus-based sediment quality guidelines for freshwater ecosystems. Arch. Environ. Contam. Toxicol..

[16] Cevik F., Goksu M.Z.L., Derici O.B. (2009). An assessment of metal pollution in surface sediments of Seyhan dam by using enrichment factor, geoaccumulation index and statistical analyses. Environ. Monit. Assess..

[17] Moeinaddini M., Esmaili Sari A., Chan A.Y.C., Taghavi S.M., Hawker D., Connell D. (2014). Source apportionment of PAHs and n-alkanes in respirable particles in Tehran, Iran by wind sector and vertical profile. Environ. Sci. Pollut. Res..

[18] Inal A., Boulahdid M., Angelleti B., Radakovitch O. (2018). Levels and ecological risk assessment of heavy metals in surface sediments of fishing grounds along Algerian coast. Mar. Pollut. Bull..

[19] Basooma A., Teunen L., Semwanga N., Bervoets L. (2021). Trace metal concentrations in the abiotic and biotic components of River Rwizi ecosystem in western Uganda, and the risks to human health. Heliyon.

[20] Xue S., Jian H., Yang F., Liu Q., Yao Q. (2022). Impact of water-sediment regulation on the concentration and transport of dissolved heavy metals in the middle and lower reaches of the Yellow River. Sci. Total Environ..

[21] Macdonald D.D., Carr R.S., Calder F.D., Long E.R., Ingersoll C.G. (1996). Development and evaluation of sediment quality guidelines for Florida coastal waters. Ecotoxicology.

[22] Long E.R., Macdonald D.D., Smith S.L., Calder F.D. (1995). Incidence of adverse biological effects within ranges of chemical concentrations in marine and estuarine sediments. Environ. Manag..

[23] Guendouzi Y., Boulahdid M., Hacene O.R., Inal A., Boudjellal B., Fowler S.W. (2021). Contamination level and ecological risk assessment of particulate trace metals in southwestern Mediterranean Sea. Reg. Stud. Mar. Sci..

[24] Zhang Y.H., Du J.M. (2005). Background values of pollutants in sediments of the South China Sea. Acta Oceanol. Sin..

[25] Hakanson L. (1980). An ecological risk index for aquatic pollution control. A sedimentological approach. Water Res..

[26] Esslemont G. (2000). Heavy metals in seawater; marine sediments and corals from the Townsville section, great barrier reef Marine Park, Queensland. Mar. Chem..

[27] Kumar V., Sinha A.K., Rodrigues P.P., Mubiana V.K., Blust R., De Boeck G. (2015). Linking environmental heavy metal concentrations and salinity gradients with metal accumulation and their effects: A case study in 3 mussel species of Vitória Estuary and Espírito Santo Bay, Southeast Brazil. Sci. Total Environ..

[28] Larrose A., Coynel A., Schäfer J., Blanc G., Massé L., Maneux E. (2010). Assessing the current state of the Gironde Estuary by mapping priority contaminant distribution and risk potential in surface sediment. Appl. Geochem..

[29] Paatero P., Tapper U. (1994). Positive matrix factorization: A non-negative factor model with optimal utilization of error estimates of data values. Environmetrics.

[30] Zhang J., Sun Y., Wu F., Sun J., Wang Y. (2014). The characteristics, seasonal variation and source apportionment of VOCs at Gongga Mountain, China. Atmos. Environ..

[31] US EPA (U.S. Environmental Protection Agency) (2014). EPA Positive Matrix Factorization (PMF) 5.0, Fundamentals and User Guide. https://www.epa.gov/heasd/research/pmf.html.

[32] Hyun S., Lee C.H., Lee T., Choi J.W. (2007). Anthropogenic contributions to heavy metal distributions in the surface sediments of Masan Bay, Korea. Mar. Pollut. Bull..

[33] Han D.M., Cheng J.P., Hu X.F., Jiang Z.Y., Mo L., Xu H., Ma Y.N., Chen X.J., Wang H.L. (2017). Spatial distribution; risk assessment and source identification of heavy metals in sediments of the Yangtze River Estuary, China. Mar. Pollut. Bull..

[34] Voulgaropoulou S., Spanos G., Angelis L. Analyzing measurements of the R statistical open source software. Proceedings of the 2012 35th Annual IEEE Software Engineering Workshop.

[35] Yan N., Liu W.B., Xie H.T., Gao L.R., Han Y., Wang M.J., Li H.F. (2016). Distribution and assessment of heavy metals in the surface sediment of Yellow River, China. Chin. J. Environ. Sci..

[36] Chen Y.Z., Yang W., Cao Y.G., Liu C.J., Li R.X. (2020). Seasonal characteristics of circulation in the northern Beibu Gulf. J. Guangdong Ocean. Univ..

[37] Zhu H., Bing H.J., Yi H.P., Wu Y.H., Sun Z.G. (2018). Spatial distribution and contamination assessment of heavy metals in surface sediments of the Caofeidian adjacent sea after the land reclamation, Bohai Bay. J. Chem..

[38] Yao W.M., Hu C.Y., Yang X.L., Shui B.N. (2021). Spatial variations and potential risks of heavy metals in sediments of Yueqing Bay, China. Mar. Pollut. Bull..

[39] Sun X., Li B.S., Liu X.L., Li C.X. (2020). Spatial variations and potential risks of heavy metals in seawater, sediments, and living organisms in Jiuzhen Bay, China. J. Chem..

[40] Wu M.L., Cheng H., Zhao H., Sun F.L., Wang Y.T., Yin J.P., Fei J., Sun C.C., Wang Y.S. (2020). Distribution patterns and source identification for heavy metals in Mirs Bay of Hong Kong in China. Ecotoxicology.

[41] Zhao G., Ye S., Yuan H., Ding X., Wang J. (2016). Distribution and contamination of heavy metals in surface sediments of the Daya Bay and adjacent shelf, China. Mar. Pollut. Bull..

[42] Li G.G., Hu B.Q., Bi J.Q., Leng Q.N., Xiao C.Q., Yang Z.C. (2013). Heavy metals distribution and contamination in surface sediments of the coastal Shandong Peninsula (Yellow Sea). Mar. Pollut. Bull..

[43] Hu B., Li G., Li J., Bi J., Zhao J., Bu R. (2013). Spatial distribution and ecotoxicological risk assessment of heavy metals in surface sediments of the southern Bohai Bay, China. Environ. Sci. Pollut. Res..

[44] Yuan H., Song J., Li X., Li N., Duan L. (2012). Distribution and contamination of heavy metals in surface sediments of the South Yellow Sea. Mar. Pollut. Bull..

[45] Liu S., Shi X., Liu Y., Zhu Z., Yang G., Zhu A., Gao J. (2011). Concentration distribution and assessment of heavy metals in sediments of mud area from inner continental shelf of the East China Sea. Environ. Earth Sci..

[46] Christophoridis C., Bourliva A., Evgenakis E., Papadopoulou L., Fytianos K. (2019). Effects of anthropogenic activities on the levels of heavy metals in marine surface sediments of the Thessaloniki Bay, Northern Greece: Spatial distribution; sources and contamination assessment. Microchem. J..

[47] Islam M.S., Ahmed M.K., Raknuzzaman M., Habibullah-Al-Mamun M., Islam M.K. (2015). Heavy metal pollution in surface water and sediment: A preliminary assessment of an urban river in a developing country. Ecol. Indic..

[48] Rahman M.S., Saha N., Molla A.H. (2014). Potential ecological risk assessment of heavy metal contamination in sediment and water body around Dhaka export processing zone, Bangladesh. Environ. Earth Sci..

[49] Jeong H., Choi J.Y., Choi D.H., Noh J.H., Ra K. (2021). Heavy metal pollution assessment in coastal sediments and bioaccumulation on seagrass (*Enhalus acoroides*) of Palau. Mar. Pollut. Bull..

[50] Fujita M., Ide Y., Sato D., Kench P.S., Kuwahara Y., Yokoki H., Kayanne H. (2014). Heavy metal contamination of coastal lagoon sediments: Fongafale Islet, Funafuti Atoll, Tuvalu. Chemosphere.

[51] Dou Y.G., Li J., Zhao J.T., Hu B.Q., Yang S.Y. (2013). Distribution; enrichment and source of heavy metals in surface sediments of the eastern Beibu Bay, South China Sea. Mar. Pollut. Bull..

[52] Tribovillard N. (2020). Arsenic in marine sediments: How robust a redox proxy?. Palaeogeogr. Palaeocl..

[53] Yang J.C., Wang W.G., Zhao M.W., Chen B., Dada Q.A., Chu Z.H. (2015). Spatial distribution and historical trends of heavy metals in the sediments of petroleum producing regions of the Beibu Gulf, China. Mar. Pollut. Bull..

[54] Xia P., Meng X., Yin P., Cao Z., Wang X. (2011). Eighty-year sedimentary record of heavy metal inputs in the intertidal sediments from the Nanliu River estuary, Beibu Gulf of South China Sea. Environ. Pollut..

[55] Gao H., Zhang Y., Zhang K. (2002). Atmospheric inputs of pollutants to the sea, their effects on marine environment and ecosystem. Adv. Earth Sci..

[56] Shikazono N., Tatewaki K., Mohiuddin K.M., Nakano T., Zakir H.M. (2012). Sources, spatial variation and speciation of heavy metals in sediments of the Tamagawa River in Central Japan. Environ. Geochem. Health.

[57] Achary M.S., Panigrahi S., Satpathy K., Prabhu R., Panigrahy R. (2016). Health risk assessment and seasonal distribution of dissolved trace metals in surface waters of Kalpakkam, southwest coast of Bay of Bengal. Regional Stud. Mar. Sci..

[58] Pravin U.S., Trivedi P., Ravindra M.M. (2012). Sediment heavy metal contaminants in Vasai Creek of Mumbai: Pollution impacts. Am. J. Chem..

[59] Bhuiyan M.A.H., Suruvi N.I., Dampare S.B., Islam M.A., Quraishi S.B., Ganyaglo S., Suzuki S. (2011). Investigation of the possible sources of heavy metal contamination in lagoon and canal water in the tannery industrial area in Dhaka, Bangladesh. Environ. Monit. Assess..

[60] Fu J., Zhao C., Luo Y., Liu C., Kyzas G.Z., Luo Y., Zhao D., An S., Zhu H. (2014). Heavy metals in surface sediments of the Jialu River, China: Their relations to environmental factors. J. Hazard. Mater..

