# Supplementary material for: Spatial Distribution, Potential Risks and Source Identification of Heavy Metals in the Coastal Sediments of the Northern Beibu Gulf, South China Sea
Source: Int J Environ Res Public Health. 2022 Aug 17;19(16):10205. doi: 10.3390/ijerph191610205 (PMC9408468; doi:10.3390/ijerph191610205)
Supplement: Supplementary file 1 [file ijerph-19-10205-s001.zip › ijerph-1796370-supplementary.pdf]

Table S1. Sediment quality (SQGs) values for each heavy metal (mg/kg).

SQG	Cr	Cu	Zn	As	Cd	Pb	Reference
TEL	52.3	18.7	124	7.24	0.68	30.2	[1]
ERM	160	108	271	41.6	4.21	112	[1]
B_i	39.3	7.43	54.4	9.71	0.18	15.6	[2]
T_r^i	2	5	1	5	30	5	[3]
MSQ-1	80	35	150	20	0.5	60	[4]

Table S2. Classification and description of sediment quality guidelines (SQGs).

Class	Category	Description
Grade I	MCs ≤ TEL	No adverse biological effects
Grade II	TEL < MCs ≤ ERM	Adverse effects occasionally occur
Grade III	MCs > ERM	Adverse effects frequently occur
Grade IV	m-ERM-Q < 0.1	9% probability of toxicity
Grade V	0.1 < m-ERM-Q ≤ 0.5	21% probability of toxicity
Grade VI	0.5 < m-ERM-Q ≤ 1.5	49% probability of toxicity
Grade VII	m-ERM-Q > 1.5	76% probability of toxicity

Notes: MCs means heavy metals; TEL means threshold effect level; ERM means effect range median; m-ERM-Q indicates mean-effect range medium-quotient.

Table S3. Classification and description of I_{geo} values for heavy metals in surface sediments.

Class	I_{geo} value	Pollution level
0	$I_{geo} \leqslant 0$	Uncontaminated
1	$0 < I_{geo} \leqslant 1$	Uncontaminated to moderately contaminated
2	$1 < I_{geo} \leqslant 2$	Moderately contaminated
3	$2 < I_{geo} \leqslant 3$	Moderately to seriously contaminated
4	$3 < I_{geo} \leqslant 4$	Seriously contaminated
5	$4 < I_{geo} \leqslant 5$	Seriously to extremely contaminated
6	$I_{geo} > 5$	Extremely contaminated

Table S4. Classification and description of ecological risk index (E_r^i) and hazard quotient index (RI).

E_r^i	Description	RI	Description
$E_r^i < 40$	Low risk	$RI < 150$	Low risk
$40 \leqslant E_r^i < 80$	Moderate risk	$150 \leqslant RI < 300$	Moderate risk
$80 \leqslant E_r^i < 160$	Considerable risk	$300 \leqslant RI < 600$	Considerable risk
$160 \leqslant E_r^i < 320$	High risk	$RI \geqslant 600$	High risk
$E_r^i \geqslant 320$	Very high risk		

Table S5. I_{geo} values for metals in the sediment samples.

Station	Cr	Cu	Zn	As	Cd	Pb
S1	-1.30	-0.09	-0.32	-0.94	-2.18	**0.02**
S2	-1.73	-0.19	-0.55	-0.68	-2.26	-0.09
S3	-1.48	-0.15	-0.49	-0.68	-2.20	0.17
S4	-2.12	0.19	-0.63	-1.11	-1.93	-0.23
S5	-1.53	-0.03	-0.54	-0.64	-2.23	-0.03
S6	-1.46	-0.29	-0.40	-0.63	-1.88	**0.05**
S7	-1.55	-0.55	-0.61	-1.33	-2.64	-0.28
S8	-3.21	-2.38	-1.63	-0.19	-3.93	-1.42
S9	-2.11	-0.97	-1.32	-1.39	-3.07	-0.79
S10	-2.20	-1.20	-1.30	-1.64	-3.19	-0.83
S11	-3.67	-3.02	-2.14	-1.92	-4.22	-2.06
S12	-2.36	-1.36	-1.47	-1.89	-2.79	-0.91
S13	-1.84	-0.72	-0.92	-1.33	-2.60	-0.55
S14	-1.45	-0.30	-0.47	-1.22	-2.23	-0.15
S15	-3.00	-2.66	-2.26	-2.04	-4.22	-0.77
S16	-3.99	-3.73	-3.25	-1.47	-5.02	-2.09
S17	-1.71	-0.59	-0.77	-1.04	-2.78	-0.39
S18	-1.42	-0.28	-0.41	-1.03	-2.58	-0.14
S19	-3.48	-3.41	-3.15	-2.33	-3.80	-2.08
S20	-1.38	-0.20	-0.38	-1.14	-2.50	-0.04
S21	-2.18	-0.79	-1.22	-1.25	-3.16	-0.53
S22	-2.18	-1.33	-1.57	-1.64	-3.47	-0.85
S23	-1.38	-0.27	-0.39	-0.04	-2.34	**0.32**
S24	-1.39	-0.28	-0.41	-0.74	-2.72	-0.06
S25	-2.48	-1.27	-1.66	-1.98	-3.60	-1.15
S26	-1.70	-0.66	-0.81	-1.05	-2.92	-0.39
S27	-2.13	-1.06	-1.38	-1.34	-3.21	-0.63
S28	-1.72	-1.36	-1.21	-0.23	-3.60	-0.10
S29	-2.73	-1.42	-1.16	-1.38	-2.45	-1.17
S30	-1.81	-0.76	-0.96	-1.02	-2.62	-0.13

Table S6. E_r^i and *RI* values for heavy metals in surface sediments of the northern Beibu Gulf.

Station	E_r^i						*RI*
	Cr	Cu	Zn	As	Cd	Pb	
S1	1.22	7.03	1.20	3.90	9.94	7.59	30.88
S2	0.90	6.58	1.02	4.68	9.40	7.04	29.62
S3	1.07	6.77	1.07	4.69	9.78	8.47	31.84
S4	0.69	8.57	0.97	3.47	11.82	6.41	31.93
S5	1.04	7.33	1.03	4.83	9.58	7.37	31.18
S6	1.09	6.13	1.14	4.84	12.20	7.78	33.19
S7	1.02	5.11	0.98	2.98	7.22	6.18	23.50
S8	0.32	1.44	0.48	6.56	2.95	2.81	14.57
S9	0.70	3.83	0.60	2.85	5.35	4.33	17.66
S10	0.65	3.27	0.61	2.41	4.92	4.21	16.07
S11	0.24	0.92	0.34	1.98	2.42	1.80	7.71
S12	0.59	2.92	0.54	2.02	6.51	4.00	16.57
S13	0.84	4.55	0.79	2.98	7.42	5.13	21.70
S14	1.10	6.09	1.08	3.22	9.57	6.76	27.83
S15	0.38	1.19	0.31	1.82	2.41	4.40	10.51
S16	0.19	0.56	0.16	2.71	1.39	1.77	6.77
S17	0.92	4.99	0.88	3.66	6.53	5.71	22.69
S18	1.12	6.19	1.13	3.67	7.52	6.79	26.42
S19	0.27	0.70	0.17	1.49	3.24	1.77	7.64
S20	1.15	6.53	1.15	3.41	7.94	7.32	27.50
S21	0.66	4.33	0.65	3.15	5.04	5.18	19.00
S22	0.66	2.99	0.51	2.40	4.05	4.15	14.76
S23	1.15	6.21	1.15	7.28	8.87	9.36	34.00
S24	1.15	6.17	1.13	4.48	6.85	7.21	26.98
S25	0.54	3.10	0.48	1.90	3.70	3.38	13.09
S26	0.92	4.76	0.85	3.62	5.94	5.71	21.81
S27	0.69	3.59	0.58	2.95	4.86	4.85	17.51
S28	0.91	2.92	0.65	6.40	3.71	7.00	21.59
S29	0.45	2.80	0.67	2.89	8.25	3.33	18.39
S30	0.85	4.42	0.77	3.69	7.33	6.88	23.94

Table S7. Pearson correlation (PC) coefficient matrix of heavy metals in the surface sediments of the northern Beibu Gulf.

	Cr	Cu	Zn	As	Cd	Pb	TOC
Cr	1.000						
Cu	0.860**	1.000					
Zn	**0.942****	0.935**	1.000				
As	0.497	0.415	0.525	1.000			
Cd	0.734*	0.894**	0.886*	0.362*	1.000		
Pb	**0.931****	0.865*	**0.905****	0.629*	0.776*	1.000	
TOC	0.775*	0.850*	0.801*	0.439*	0.830**	0.827*	1.000

* $p < 0.05$ ** $p < 0.01$.

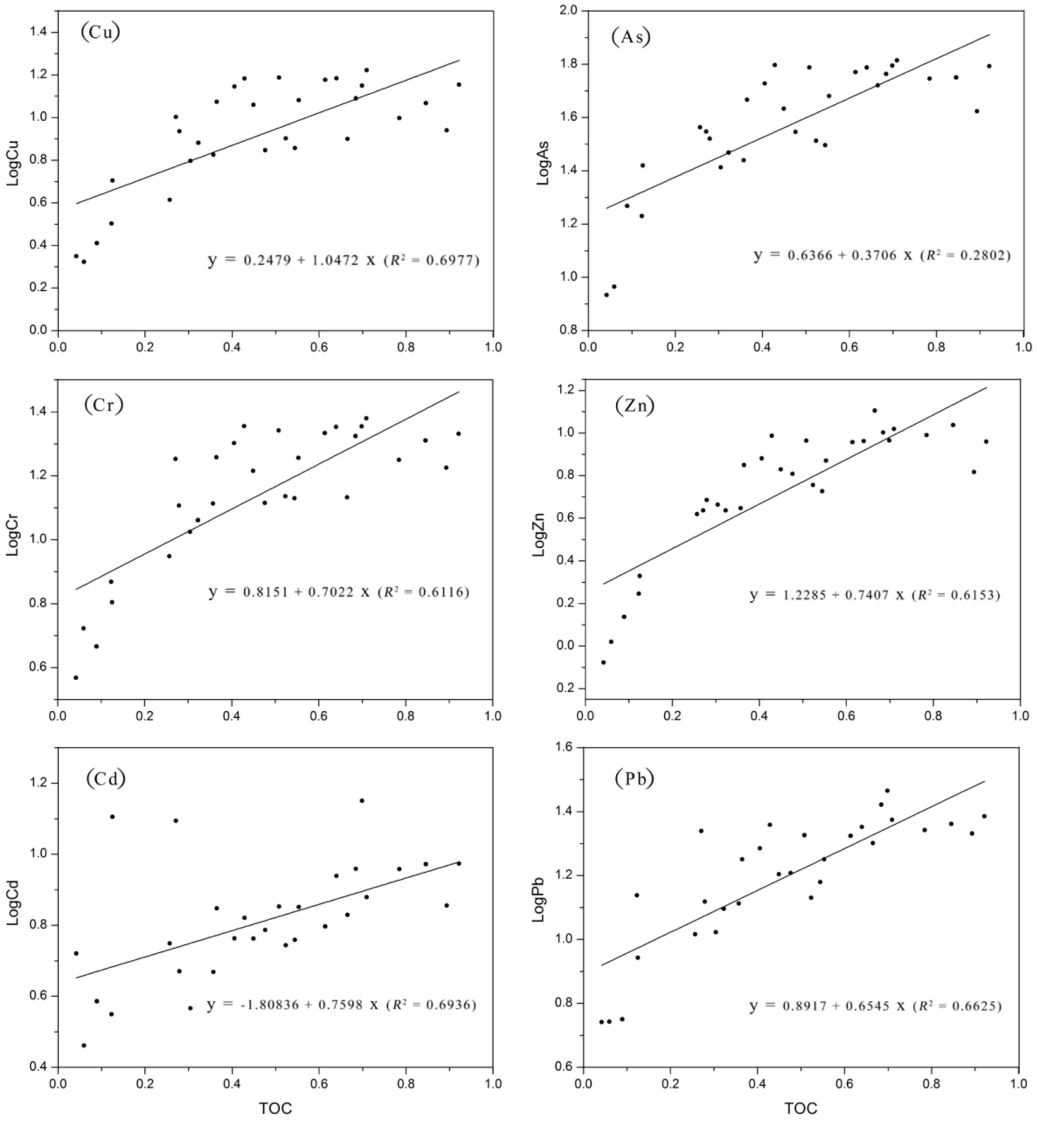

Figure S1. Linear regression of TOC contents versus logarithm transformed metal concentrations in sediments of the northern Beibu Gulf.

References

1. Macdonald, D.D.; Carr, R.S.; Calder, F.D.; Long, E.R.; Ingersoll, C.G. Development and evaluation of sediment quality guidelines for Florida coastal waters. Ecotoxicology, 1996, 5: 253–278.
2. Zhang, Y.H.; Du, J.M. Background values of pollutants in sediments of the South China Sea. Acta Oceanol. Sin. 2005, 27: 161–166. (In Chinese)
3. Larrose, A.; Coynel, A.; Schäfer, J.; Blanc, G.; Massé, L.; Maneux, E. Assessing the current state of the Gironde Estuary by mapping priority contaminant distribution and risk potential in surface sediment. Appl. Geochem. 2010, 25: 1912–1923.
4. Yao, W.M.; Hu, C.Y.; Yang, X.L.; Shui, B.N. Spatial variations and potential risks of heavy metals in sediments of Yueqing Bay, China. Mar. Pollut. Bull. 2021, 173: 112983.
